# On the Treatment of Blennorrhagic Affections by Gurjun Balsam

**Published:** 1878-09

**Authors:** E. Vidal

**Affiliations:** Paris, France, Physician to the Hospital of Saint Louis


### ON THE TREATMENT OF BLENNORRHAGIC AFFECTIONS BY GURJUN BALSAM.

By Dr. E. VIDAL, Paris, France,
Physician to the Hospital of Saint Louis.

*Translated, with the Consent of the Author,*
By R. J. NUNN, M.D.

About a year ago, having received into my wards, at the Hospital of St. Louis, a woman about thirty years of age, affected with leprosy of the tuberculous and anesthetic variety, it occurred to me to treat her with Gurjun balsam or wood oil, an oleo-resin obtained from a tree of the family of depterocarpees, which had already been successfully employed in the treatment of leprosy by Dr. Joseph Dougall, surgeon-major of the medical service of Madras.

The flattering results obtained in 1873 in the Haddo leper hospital in the islands of Andaman caused this remedy to be officially recommended by the organ of the Marquis of Salisbury, in a circular addressed by the English government to the physicians of the Indian empire; and finally it has been highly praised by that celebrated dermatologist, Erasmus Wilson, who has experimented with it in London for the treatment of leprosy, of certain ulcerative affections of the skin, and of some forms of eczema, etc. etc.

At my request, a distinguished apothecary of Paris, Mr. A. Petit, procured for me from England a quantity of the balsam of Gurjun, and about the first of October, 1876, I commenced the medication of my patient. I began with a drachm (4 grammes) in emulsion as a draught, and I gradually increased the dose to a drachm and three quarters (7 grammes). Inunctions were also made night and morning over the leprous tubercules with a liniment composed of equal parts of lime water and Gurjun balsam. This treatment was followed by notable improvement. Continued, after leaving the hospital, in the daily dose of one drachm (4 grammes), combined with the inunctions, it effected a cure almost complete—that is, if I may credit a letter which I received June 3d, 1877; but in spite of the rather enthusiastic assertions of the patient, I am not yet prepared to admit more than an improvement, knowing how little reliance is to be placed on the transient remissions, the false appearances of cure in leprosy.

Dr. Dougall, who first conceived the idea of treating leprosy with Gurjun balsam, in his report on that subject, says that the medicine in question is commonly employed by the Benglese as a remedy for blennorrhagia. Previous to this, in 1838, Sir William O'Shanghnessy, of the Indian army, had suggested Gurjun balsam as an important succedaneum for copaiva, and even considered it preferable. Favorable results were also obtained in it by Dr. E. J. Waring (1852), Dr. Montgomery (1862), Dr. Henderson (1865), Drs. Rean, Kanny-Loll-Dey, Coulson, and Sir Ronald Martin.

Wishing to assure myself of the efficacy of this medicine, I have administered it in fifteen cases of urethral blennorrhagia—thirteen males and two females—with the following therapeutic effects:

After forty-eight hours there was a very marked alleviation of symptoms, an almost complete cessation of the pain which accompanied micturition, an increase of urinary secretion, and a very considerable diminution of the discharge, the color of which was already remarkably changed. From green or yellow it had assumed a whitish color, and had become of a rather thickish consistence.

On the sixth or eighth day the blennorrhagia had entirely ceased in the more favorable cases; in the others there was not more than a slight oozing in the morning, and by the tenth or twentieth day the cure was complete. It was delayed, however, to the twenty-fourth day in the case of a man to whom the medicine was administered in the acute stage, and whose treatment was further complicated by an attack of double epididymitis.

The most rapid cures affected were in cases of old discharges, blennorrhœas and blennorrhagias at the period of full development, when, the acute stage being passed, the pain during micturition had diminished, and the meatus was no longer red nor swollen.

According to experience, this is the stage of the disease which should be awaited before commencing the use of balsams. It is at this time that their action is truly efficacious; their earlier administration entails the risk of obtaining less favorable results, of retarding the cure, or even of doing injury.

Although in this regard I share the opinion to which all experimenters have arrived, still I infringed upon the rule, and to five cases I ordered the Gurjun balsam during the more acute period of blennorrhagia. In one of these cases, attacked with chordee, a senseless effort on the part of the patient to violently straighten the penis brought on a traumatic hematuria, which compelled me to stop the balsam and adopt vigorous antiphogistic measures. In the four others there was remarkable improvement from the beginning, a cessation of pain after forty-eight or seventy-two hours, and cure on the fifteenth or twentieth day of treatment.

The observations on blennorrhagia which I have made at the hospital have been collected by my interus, Messrs. Darcy and de Beauman, and recorded in an inaugural thesis, sustained some days since by one of my pupils, Dr. Luc Deval.

*Mode of Employment.*—Sir William O'Shanghnessy and Edward John Waring administered the Gurjun balsam in very small doses, ten to twenty drops a day. Dr. Henderson prescribed two to three teaspoonfuls. The dose which appears to me to be sufficient is a teaspoonful (4 grammes) daily. I have rarely exceeded one and one-half teaspoonful (6 grammes). Larger doses produce gastro-intestinal intolerance.

Gurjun balsam is not solidified by magnesia like copaiva. The pure balsam, put up in gelatine or gluten capsules, has been less easily tolerated than the following emulsion:

℞.—Gurjun balsam, . . ʒi (4 grammes.)

Gum, . . . . . ʒi (4 " )

Syrup (simple or of cachon, ʒiij (12 " )

Infusion of . . . ℥iʒiij(40 " )

Make an emulsion.

This mixture should be taken in two doses, one-half before breakfast, the other portion before dinner, *just before the meal.*

Having observed that the patients to whom I permitted the use of wine tolerated the Gurjun balsam well, experiencing neither nausea nor pains in the stomach, and recovering as quickly as the others, I prescribe a liquor glass of Malaga wine, or of Bagnols, to be taken immediately after the medicine. If these wines cannot be had, a quarter of a glass of red wine answers just as well; and further, I allow the regular wine ration during the meal.

Let the Gurjun balsam be taken immediately before eating, and facilitate its digestion by a little wine. Such is my experience on the necessary conditions to cause it to be easily tolerated.

A mode of administration not so good, and the employment of too strong doses, are probably the reasons which have prevented this excellent anti-blennorrhagic from becoming popular in England, as it ought to be, after the researches of the distinguished physicians who have praised it.

Even given alone, without alcohol, the Gurjun balsam is better borne than copaiba. It produces in the stomach a sensation of warmth; sometimes a little nausea, and vomiting is very exceptional. It produces one, rarely two semiconsistent alvine evacuations within two hours after the meal, but in doses larger than one and a half teaspoonfuls (6 grammes) it may cause colics and diarrhœa.

In none of my patients did it give rise to an erythemato-papulous eruption such as is sometimes occasioned by copaiba. On the contrary, a patient recently under my charge, affected with the roseola of copaiba, was quickly cured of his blennorrhœa without the eruption reappearing. I do not think that Dr. Maurice or Dr. Mallery, who, since I commenced this investigation have prescribed the Gurjun balsam in some cases of blennorrhagia, have observed any eruptions produced by this medicine. Dr. Montgomery, quoted by Erasmus Wilson, is, to my knowledge, the only one who has observed a case of erythema analogus to that which may follow the use of copaiba.

The taste of Gurjun balsam is less disagreeable than that of copaiba or cubebs, and it communicates to the breath and the urin an odor more delicate than those substances.

The urine has a feeble balsamique odor, not disagreeable, which is quickly dissipated. One part of the oleoresin is eliminated by the urine as has been analytically demonstrated by Mr. Monin, pharmacun to my department.

Among the salts contained in the urine are some which present the characters of alkaline copaibates, but their acids have not been well determined. They are formed by the resinous acids of the Gurjun, combined with the alkaline bases of the urine.

After having experimented with the Gurjun balsam in the treatment of urethritis, I determined to employ it as a topical application in other blennorrhagic affections. In the form of a liniment it rapidly arrested the inflamation and stopped the discharge in two cases of balanitis and three cases of vaginitis.

The cases of vaginitis were cured in six or eight days; they were treated by first injecting with warm water and then applying, by means of a speculum, a tampon of cotton, wet with the liniment, a second tampon of dry cotton, attached to the first by a thread, sewed to maintain it in place, and this dressing has not been made oftener than once a day. The first application causes a little smarting, the second produces less heat, and the following days in proportion as the cure progresses the sensation of warmth occasioned by the liniment diminishes.

After the first dressing the discharge was modified and by the third or fourth day, it was nearly dried up.

The liniment is composed of equal parts of Gurjun balsam and lime water. These proportions, already given by Dr. Dougall and by Dr. Erasmus Wilson, are the only ones which will produce a good emulsion.

To those more competent than myself, I leave the pharmaceutical study of Gurjun balsam. It seems to have been already pushed very far by the works of Charles Low (in Pharmaceutical Journal and Transactions, 1854, p. 65,) of Daniel Hanburg, 1856, of Vry, of Rotterdam, 1857, of Guibourt, of Fluckiger, 1866, of Huseman, of Planchon. This substance is mentioned in all the new treaties on materia medica, or pharmacology.

In all it is said that the extreme cheapness of that oleoresin explains why it is often employed to adulterate balsam copaiba.

In India, the production of Gurjun balsam, or wood oil, is so considerable, and its value so little, that it is employed to paint the wood work of houses and vessels.

It is obtained by incising the bark of large trees of the family of the dipterocarpies. These trees, remarkable for the beauty of form and for their height, which is often seventy yards, grow in the islands of the Indian Archipelago, and in the countries east of Bengal. Roxburgh says that a single tree of the "dipterocarpus turbinatus" can furnish in one season a crop of as much as forty gallons of wood oil.

